# Affect, Body, and Eating Habits in Children: A Systematic Review

**DOI:** 10.3390/nu15153343

**Published:** 2023-07-27

**Authors:** Marzieh Abdoli, Marco Scotto Rosato, Annarosa Cipriano, Rosanna Napolano, Paolo Cotrufo, Nadia Barberis, Stefania Cella

**Affiliations:** 1 Observatory on Eating Disorders, Department of Psychology, University of Campania “Luigi Vanvitelli”, Viale Ellittico, 31, 81100 Caserta, Italy; marzieh.abdoli@unicampania.it (M.A.); marco.scottorosato@unicampania.it (M.S.R.); annarosa.cipriano@unicampania.it (A.C.); rosanna.napolano@studenti.unicampania.it (R.N.); paolo.cotrufo@unicampania.it (P.C.); 2 Department of Medical and Surgical Sciences, University “Magna Graecia” of Catanzaro, 88100 Catanzaro, Italy; nbarberis@unicz.it

**Keywords:** eating habits, emotions, affect, body, children

## Abstract

The present review investigates the complex associations between children’s affective states, body perceptions, and eating habits, thus providing crucial insights for potential health interventions. Following PRISMA guidelines, three databases were searched for peer-reviewed studies exploring the relationship between eating habits, emotional states, and body image perceptions in a population of children (5 to 11 years old). A total of seven articles were included. Our findings revealed a pattern of associations between negative emotional states, like anxiety and depressive feelings, and maladaptive eating behaviors. Additionally, explicit influences from parental feeding practices, peer pressure, socioeconomic factors, and children’s body perceptions were observed to shape eating habits, with a pronounced tendency among older girls towards dieting and food preoccupation. Our results underline the intertwining nature of age, gender, and emotional states. Furthermore, our findings accentuate the urgency for comprehensive interventions that acknowledge and address the complex interplay of emotional, familial, and socioeconomic factors alongside children’s body image perceptions. The criticality of continued research, particularly ones employing longitudinal designs and diverse demographic samples, is highlighted as we strive to understand and navigate such multifaceted relationships to enhance children’s health and well-being.

## 1. Introduction

Eating habits are vital to children’s health and well-being, potentially impacting physical, emotional, and psychological development [1,2,3]. Given the exceeding worldwide occurrence of childhood obesity and related health issues, the relevance and urgency of understanding children’s eating habits has become paramount. Studies have found that the prevalence of unhealthy eating habits and obesity in children is alarmingly high, with significant long-term consequences [4,5]. Engaging in unhealthy eating habits during childhood can set a trajectory for adverse health outcomes, including obesity, diabetes, and cardiovascular diseases well into adulthood [6,7,8]. The prevalence of childhood obesity has been connected to unhealthy dietary habits, with high consumption of energy-dense and nutrient-poor foods and a low intake of fruits and vegetables [9]. Furthermore, the consequences of such unhealthy eating habits extend beyond physical health, impacting mental health, cognitive development, academic performance, and emotional well-being [10,11]. Healthy childhood eating habits are crucial as they create the foundation for long-term dietary patterns, influencing the threat of developing diseases like obesity, diabetes, and cardiovascular problems later in life [4,5,12,13]. Beyond biological, social, and environmental factors, emotions have increasingly been recognized for their role in shaping children’s eating habits [14,15,16,17,18]. The importance of emotions in our lives cannot be understated. Emotions shape our experiences, guide our actions, and fundamentally influence our well-being. In the context of eating habits, the theory proposed by psychosomatic pioneer Hilde Bruch has led to an expanded understanding of the role of emotions [19]. Bruch emphasized the crucial role of maternal attitudes during infancy; a mother’s inability to respond empathetically to a child’s emotional needs profoundly impacts the child’s relationship with food [19]. This potentially leads to the child resorting to food as an emotional regulator, thereby blurring the lines between emotional and physical needs [20]. A solid sense of self and body integrity is essential for healthy emotional development and eating behaviors [21]. The relationship between emotions and eating habits is supported both theoretically and empirically. Research has shown that positive and negative emotions greatly influence food choices and eating behaviors, particularly in children. Moreover, negative emotions like stress, anxiety, and sadness have been associated with unhealthy eating habits, such as emotional eating, binge eating, and consumption of calorie-dense, nutrient-poor foods [15,22,23]. Conversely, the influence of positive emotions, like happiness and contentment, has been linked with healthier eating habits, such as increased fruit and vegetable consumption and balanced meal patterns [24,25]. Understanding the impact of emotions on children’s eating habits is crucial for shedding light on the complex interplay between psychological and physical health. This is especially important since children are at an increased risk for eating disorders (EDs) [26,27,28]. Emotional eating, i.e., eating in response to emotional cues rather than physiological hunger, has been associated with developing obesity and eating disorders in childhood [29,30,31,32]. Food is often used as an emotional regulator, providing comfort in the form of high-calorie, palatable foods during challenging times. Over time, this behavior can reinforce unhealthy eating patterns [31,33,34]. Additionally, the emotional experiences of a child can influence their perception of hunger and satiety, disrupting eating patterns and food choices [35,36,37]. Research has increasingly focused on understanding the connection between emotions and eating habits in children [38,39]. For instance, Goldschmidt and colleagues explored the relationships between emotional regulation, dietary restraint, and unhealthy eating in children, demonstrating a significant connection [40]. Similarly, a longitudinal study by Van Strien and colleagues [41] found that emotional eating in children increased over time in response to stress. These studies have predominantly centered on the role of negative emotions, with less emphasis on the influence of positive emotions [42,43,44,45,46,47]. Given the significant public health implications of understanding the relationship between emotions and eating habits, a comprehensive synthesis of the available evidence is needed to inform future research and interventions. This systematic review aims to provide an exhaustive overview of scientific literature on the relationship between emotions and eating habits in children aged 5 to 11. We sought to identify patterns and trends in existing research and spotlight areas for future investigation, thus contributing to a more practical understanding of the role of emotions in shaping children’s eating habits. In turn, the findings could inform the development of targeted interventions to promote healthier eating habits among this vulnerable age group.

## 2. Materials and Methods

### 2.1. Study Protocol

This study was carried out in accordance with the updated Preferred Reporting Items for Systematic Reviews and Meta-Analysis (PRISMA) guidelines [48]. A systematic search was performed using the following databases: PubMed, Scopus, and Web of Science. The research string included the following keywords: “Affect”, “Affect regulation”, “Affect dysregulation”, “Negative affect”, “Affect recognition”, “Alexithymia”, “Anxiety”, “Depression”, “Emotional awareness”, “Emotional control”, “Emotional dysregulation”, “Emotional expressi*”, “Emotional modulation”, “Emotional regulation”, “Mood”, “Child”, “Weight control”, “Weight management”, “Weight modulation”, “External eating”, “Binge eating”, “Affective eating”, “Body mass index”, “BMI”, “Eating attitude”, “Eating behavior”, “Eating habit”, “Eating practice”, “Eating style”, “Emotional eating”, and no filters or restrictions were used (Table 1). The searches were supplemented with reference lists of pertinent articles. The review protocol has been registered in the PROSPERO database (CRD42023442260).

### 2.2. Eligibility Criteria

The following eligibility criteria were adopted: (a) original peer-reviewed research conducted on the general or clinical population published in English; (b) studies in which children reported measures related to nutrition and emotions or affect; (c) studies on a sample of children between the ages of 5 and 11; and (d) children did not have any other medical conditions. Moreover, the exclusion criteria were as follows: (a) articles published in a language other than English; (b) reviews, conference papers, books, chapters, and commentaries; (c) studies conducted on toddlers, adolescents, and adults; (d) parent’s report studies; and (e) studies conducted on non-human samples. No restrictions were applied related to the type of intervention or exposure used, nor was a study required to indicate a control group. Regarding outcomes, the variables of interest were those related to feeding, emotion, and/or affect regulation, such as binge eating, emotional eating, external eating, restrained eating, eating disorder risk, mood, depression, and anxiety. Additionally, descriptive measures, such as nationality, age, BMI, and gender, have been used.

### 2.3. Study Selection

**Figure 1:**
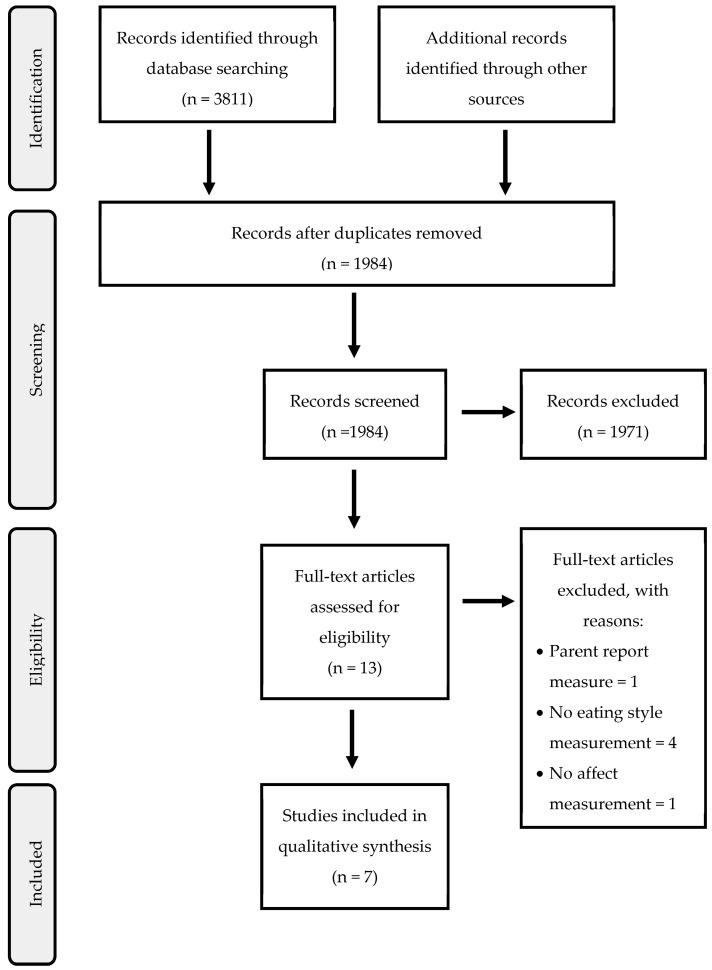
PRISMA flow diagram.

After removing duplicates using Zotero (v. 6.0.26) as reference management software, a two-stage screening process was used to select articles for this systematic review. Article selection was carried out independently by two teams of researchers (with two researchers in each group), and disagreements were resolved by the supervisor. The search was carried out on 7 March 2023. An initial selection of results was made using the search criteria with reference to the title and abstract, and the participants and procedure section if the age of the participants was not stated in the title or abstract of the articles. Articles whose title and abstract met the inclusion criteria in this first stage were eligible for a full reading of the articles. The team held regular meetings to address uncertainties and clarify the eligibility criteria. Selection discrepancies were discussed with the whole review team and resolved by the supervisor (Figure 1).

### 2.4. Assessment of Quality

The methodological quality of all included studies was assessed using the Mixed Methods Appraisal Tool (MMAT) [49]. This tool is applicable for quantitative, qualitative, and mixed methods empirical studies. Two independent reviewers assessed the quality of each study, and any disagreements were resolved through discussion and consensus or by consulting a third reviewer for final evaluation. The checklist consisted of two initial questions (qualifying criteria) applicable to all study designs and five quality questions related to the type of study (e.g., “Is the sampling strategy relevant to address the research question?”; “Is the sample representative of the target population?”). Each criterion is assessable as “yes”, “no”, or “couldn’t say”, and the methods for recognizing the study type to refer to and item specification are described in the instrument manual. According to Hong and colleagues [49], proceeding with the evaluation of articles may be inappropriate when one or both qualifying criteria are not answered affirmatively. Finally, for each study, only the questions relevant to each study design were evaluated.

### 2.5. Synthesis and Analysis of Data

Data from eligible studies were extracted into a standardized data collecting form by the two couple of researchers, and the supervisor arbitrated any differences. The following information was taken from each of the included studies: ascribing the heterogeneity of the included studies regarding study design, population, and outcome measures. Subsequently, a narrative synthesis was conducted to summarize the findings on the relationship between children’s eating behaviors, children’s bodies, emotions/affect, and identified patterns and trends in the results. In addition, variables, such as age range, gender, economic status, and types of emotion (e.g., positive or negative emotions) or affect, were considered to further deepen the relationship between eating habits and emotions/affect during childhood. Finally, we also evaluated the strength and consistency of the evidence across the studies included. To ensure that the systematic review was conducted rigorously and transparently, we heeded the PRISMA guidelines throughout the review process [49].

## 3. Results

### 3.1. Study Characteristics

Our systematic review included seven studies from 2002 to January 2018, adopting various research methodologies. Two studies utilized cross-sectional designs [50,51], two implemented comparative designs [52,53], and one study combined a cross-sectional with a longitudinal design [54]. A between-group experimental design study [55] and a population-based survey were also incorporated [56]. The subjects of investigation across these studies ranged from eating measures to emotional indices, including BMI, with the majority (six) of the studies examining all three aspects. In addition, the research in this field was conducted across multiple countries consisting of Australia, the USA, the UK, and Canada. Study characteristics are summarized in Table 2.

### 3.2. Sample Characteristics

The sample size varied across the studies, with a minimum of 154 to a maximum of 356 participants. However, Kirk and colleagues distinguished themselves by conducting an expansive population-based study involving nearly six thousand students [56]. These studies have ensured gender equity in their distribution. Most research involved first-grade students, except for two studies that used alternative recruitment methods [53,55]. The age range of participants varied, but each study included students aged eight to nine (Table 2). Farrow and colleagues additionally investigated gender differences [50].

### 3.3. Assessments of Risk of Bias

A bias assessment of the included studies was performed using MMAT [49]. All studies were included, and none were excluded based on the quality assessment. According to Crowe and colleagues [57], it is not advisable to make a numerical summary of the results because it would not provide information about which aspects of the studies involved may be problematic, potentially hiding serious defects as well. All studies passed the screening questions. Regarding the specific criteria by study type, the question that differentiated the quantitative descriptive studies was related to the risk of nonresponse bias, which, for three articles [50,51,52], could not be answered. The remaining questions, for these studies as well as for the other descriptive qualitative studies, were all successful. There was only a single article with a quantitative randomized controlled trial design [52], and the criteria regarding randomization and blinding of assessors could not be answered for this study. The detailed table with all criteria is available in the Supplementary Materials.

### 3.4. Affect, Body, and Eating Habit

The research presented by Holt and Ricciardelli offers valuable insights [52]. Notably, boys experiencing negative affect displayed a heightened vulnerability to binge eating and preoccupation with food. This susceptibility appeared to be fueled further by social pressures to eat. Interestingly, a positive affect emerged as a potential safeguard against these tendencies. Moreover, when examining girls’ eating habits, the influence of body mass index (BMI) becomes salient. Girls with higher BMIs exhibited an increased propensity for dieting, a stronger preoccupation with muscle mass, heightened body dissatisfaction, and were more likely to report experiencing negative affect [52,54]. In an inverse relationship, girls with lower BMI scores were found to be significantly influenced by societal pressures to eat. The interplay of social context and anxiety also shapes children’s eating habits. Evidence from our study suggests that children in the presence of peers demonstrating high food restriction behaviors tend to resort to external eating practices, especially during periods of moderate or high anxiety [50]. Age adds another layer of complexity to these patterns. As noted by the observations from Saling and colleagues [54], an upward trajectory in dieting and preoccupation with food becomes evident with advancing age, which is particularly pronounced among girls.

### 3.5. Secondary Outcomes

Parental influence also emerged as a salient factor. Parents’ pressure to eat was associated with increased emotional eating, poor food choices, and overeating among children [52]. Also, restrictive feeding practices contributed to emotional overeating [50,54]. Houldcroft and colleagues [51] reported a link between children’s perceptions of their parents’ feeding practices and their anxiety, depression, and eating behaviors. In addition, children hailing from lower socioeconomic backgrounds exhibited a poorer diet quality, a higher BMI, and worse psychosocial outcomes with a higher propensity towards emotional and external behaviors [56]. Specifically, Kirk and colleagues [56] demonstrated that food insecurity (lower socioeconomic backgrounds with fewer resources to purchase food) is associated with higher energy intake, higher BMI, and lower quality of diet (i.e., variety and adequacy). Also, children from lower socioeconomic backgrounds were significantly more likely to report relationship, mood, or externalizing problems [56]. Moreover, peer influence has emerged as a significant secondary outcome. Studies found that children’s eating behaviors and attitudes were significantly associated with those of their friendship groups. Dietary restraint levels within friendship groups significantly correlated with children’s maladaptive eating behaviors [50]. Another notable secondary outcome was the influence of age and gender on children’s eating behaviors. Older girls were more likely to engage in dieting and food preoccupation [54]. This reinforces the understanding that the intersectionality of age, gender, and emotional states shapes children’s eating patterns. Body dissatisfaction was another secondary outcome influencing children’s eating behaviors. Children demonstrating high body dissatisfaction were found to be more susceptible to engage in external and emotional eating [50]. This highlights the critical interplay of body image perception, emotional states, and eating behaviors among children.

## 4. Discussion

This systematic review explores the intricate links between affective states, bodily perceptions, and eating behaviors among children aged 5 to 11. Understanding such relationships is crucial due to the significant impact on children’s overall well-being and mental health [58]. Our findings carry a practical significance for parents, educators, and clinicians to tailor interventions promoting healthier habits in children. Affective states encompassing a range of emotions, both negative, such as anxiety, depression, and sadness [59], and positive, including happiness, excitement, and contentment [60], are shown to have a crucial role in the modulation of children’s eating habits and body image perceptions. For instance, negative affective states have been linked with higher emotional eating and food preoccupation, especially among boys [52]. On the other hand, body weight, height, and composition exert an undeniable influence on children’s eating habits and emotional states [61,62,63]. Our review also shows how children with higher BMIs are susceptible to experiencing negative affective states, engaging in unhealthy eating behaviors and exhibiting body dissatisfaction [52,54,64]. Eating habits, such as food choices, meal patterns, and eating styles, significantly affect children’s physical and mental health, weight status, and overall development [65,66]. For example, Tan and Holub [55] explained how negative emotions might drive unhealthy food consumption, reinforcing the salience of eating habits in children’s well-being. Furthermore, the review briefly highlights socioeconomic disparities in children’s eating behaviors, mood, and BMI. Indeed, children from lower socioeconomic backgrounds were found to have a higher engagement in emotional and externalized behaviors [56]. Research has demonstrated that socioeconomic variables affect nutrition and obesity risk due to the limited resources available to purchase food as energy-dense foods are more affordable than fruits and vegetables [67]. Indeed, trying to cut back on food expenses may paradoxically result in choosing high energy food, consuming more calories, and increasing the risk of becoming overweight and obese [68,69]. Also, the associations between socioeconomic status and eating behaviors and the degree to which the various socioeconomic status indicators were related to children’s diets show significant cultural variation, both cross-national and regional [67]. However, a more detailed exploration is required to comprehend the relationship between socioeconomic background and eating habits in children. Our findings also highlight the vital role of parental influence on children’s eating habits, particularly the connection between perceived parental pressure to eat and children’s anxiety and depression levels [51]. Our findings align with the existing empirical literature and emphasize the critical role that feeding practices and behaviors play in influencing children’s eating habits [70]. However, despite the recognized importance of the parent–child relationship in influencing many aspects of the child’s development, including eating behaviors, little attention has been paid to exploring the emotional dynamics within such relationships and how they affect children’s eating habits. According to Bruch’s psychosomatic theory [21], children’s eating habits are intricately negotiated within the context of the mother–child relationship, where food has a symbolic significance and may become a means to manage emotional experiences [71]. Notwithstanding, this area remains understudied and warrants greater attention. To fully understand the underlying dynamics influencing the development of children’s eating habits, research should move beyond the straightforward behavioral aspect of the parent–child bond (i.e., feeding practices) and explore the complex interplay between affective states and emotions surrounding the parent–child interaction. Further investigation of dynamics that may influence the relationship between affective states and eating habits is warranted.

Moreover, our results highlight that negative emotions are strictly related to dysfunctional eating habits, also suggesting a difficulty in emotion regulation abilities. Theorists and researchers agree that disordered eating results from a deficit in self-regulation and the interactive regulation of emotional states [14,72,73]. Also, difficulties in emotion regulation may activate and support a vicious cycle as they have the potential to amplify negative emotions that, in turn, are implicated in the development of new maladaptive strategies [74].

The need for more diverse samples in future research is essential. The exploration of how cultural and socioeconomic factors can influence the relationship between affective states and eating habits in children from different backgrounds would help obtain a more comprehensive and applicable understanding of diverse populations.

The complexity and significance of our findings reiterate the need for nuanced, multi-faceted interventions that consider emotional, individual, familial, and socioeconomic aspects. The identified relationships underline the importance of gender-sensitive interventions and the need to focus on the difficulties children from low-income families face. Overall, this review serves as an initial step towards fostering a comprehensive understanding of the psychological and social factors shaping children’s eating habits and body perceptions, thereby paving the way for more effective evidence-based interventions for fostering healthier eating habits and emotional well-being. The multifaceted influences shaping children’s eating habits underscore the issue’s complexity. Significant correlations were noted between affective states, parental feeding practices, BMI, anxiety, and depression; these factors all play critical roles in influencing children’s eating patterns. The importance of this interplay of factors necessitates a comprehensive approach toward promoting healthy eating behaviors among children.

### Limitations and Future Directions

This research paper is subject to limitations that should be acknowledged. Initially, the intention was to undertake a meta-analysis, but the broad age range in many studies deemed them unfit for inclusion. In addition, the selected studies, despite having adequate sample sizes and balanced gender distribution, were primarily cross-sectional, with only Saling and colleagues implementing a longitudinal design and Tan and Holub examining pre–post experimental conditions in a randomized trial [54,55]. Additionally, the research parameters excluded books, chapters, conferences, and articles not yet published or published in languages other than English. This practice may have resulted in the omission of pertinent material. Furthermore, restricting the database search to PubMed, Scopus, and Web of Science could have limited the number of eligible articles for review. Moreover, the restricted cultural and geographic diversity of the studies limits the generalizability of the results to other countries and populations. Cultural variations in emotional experiences and associated eating patterns might exist [75]. Thus, our results should be interpreted with caution. One notable limitation was our focus on self-report studies. While self-reports are a practical and commonly used method for collecting data on affect and eating habits, they are also subject to recall and response bias, which may distort the participants’ actual eating patterns and emotional states. The reliance on self-reported data also restricted the pool of eligible studies as not many self-report studies fit our inclusion criteria.

Future research should concentrate on several critical areas to address this systematic review’s limitations. More longitudinal studies and randomized controlled trials are necessary to clarify the causal relationship between affect and dysfunctional eating habits among children and preadolescents. This would lead to more definitive conclusions and provide evidence supporting or refuting the efficacy of interventions targeting affect and emotions. Future investigations should also encompass broader age ranges and more diverse samples, including children from varying cultural and socioeconomic backgrounds. This will facilitate generalizing the current review’s findings to assorted populations.

Additionally, exploring potential moderating and mediating factors, such as social support, family environment, and coping strategies, that may influence the relationship between affect and eating habits is warranted. Comprehending these factors could inform the design of more effective intervention programs. Finally, subsequent systematic reviews and meta-analyses should widen their literature searches to include articles published in languages other than English and employ more databases. This would ensure a more comprehensive representation of the relevant literature and potentially result in more robust conclusions. By addressing these areas of future research, we can enhance our understanding of the intricate relationship between affect and dysfunctional eating habits in children and preadolescents.

## 5. Conclusions

The studies that met the eligibility criteria and were analyzed in the present systematic review showed a significant association between affect or emotion and dysfunctional eating habits in populations of both normal weight and overweight or obese children. However, despite the use of inclusive keywords, few studies passed the exclusion and inclusion criteria. This observation evidences a gap in the literature: there is a small number of studies and interventions related to the factors of interest in this population. One of the criteria that may have most influenced the selection outcome is the exclusion of parent report studies. This review provides an up-to-date picture of the literature and it intends to help further research in reaching new conclusions that may help promote interventions aimed at reducing eating habits associated with weight gain. Early interventions and prevention programs in this age group could play a critical role in reducing the trend of increasing prevalence of eating habits and disorders. These interventions, moreover, should aim at vulnerable factors, such as the ability to recognize negative affect and emotions and feelings and to regulate them toward one’s body.

## Figures and Tables

**Table 1:** Database, records found, and syntax used for the literature search.

Database	Records	Syntax
PubMed	986	(“Affect regulation” [Title/Abstract] OR “Affect dysregulation” [Title/Abstract] OR “Affect recognition” [Title/Abstract] OR “Alexithymia” [Title/Abstract] OR “Anxiety” [Title/Abstract] OR “Depression” [Title/Abstract] OR “Emotional awareness” [Title/Abstract] OR “Emotional control” [Title/Abstract] OR “Emotional dysregulation” [Title/Abstract] OR “Emotional expressi*” [Title/Abstract] OR “Emotional modulation” [Title/Abstract] OR “Emotional regulation” [Title/Abstract] OR “Mood” [Title/Abstract]) AND (“Obesity” [Title/Abstract] OR “Overweight” [Title/Abstract]) AND (“Child*” [Title/Abstract]) AND (“Weight control” [Title/Abstract] OR “Weight management” [Title/Abstract] OR “Weight modulation” [Title/Abstract] OR “External eating” [Title/Abstract] OR “Binge eating” [Title/Abstract] OR “Affective eating” [Title/Abstract] OR “Body mass index” [Title/Abstract] OR “BMI” [Title/Abstract] OR “Eating attitude” [Title/Abstract] OR “Eating behavior” [Title/Abstract] OR “Eating habit” [Title/Abstract] OR “Eating practice” [Title/Abstract] OR “Eating style” [Title/Abstract] OR “Emotional eating” [Title/Abstract])
Scopus	1454	TITLE-ABS (“Affect regulation” OR “Affect dysregulation” OR “Affect recognition” OR “Alexithymia” OR “Anxiety” OR “Depression” OR “Emotional awareness” OR “Emotional control” OR “Emotional dysregulation” OR “emotional expressi*” OR “Emotional modulation” OR “Emotional regulation” OR “Mood”) AND (“Obesity” OR “Overweight”) AND (“child*”) AND (“Weight control” OR “Weight management” OR “Weight modulation” OR “External eating” OR “Binge eating” OR “Affective eating” OR “Body mass index” OR “BMI” OR “Eating attitude” OR “Eating behavior” OR “Eating habit” OR “Eating practice” OR “Eating style” OR “Emotional eating”)
Web of Science	1371	TI = (“Affect regulation” OR “Affect dysregulation” OR “Affect recognition” OR “Alexithymia” OR “Anxiety” OR “Depression” OR “Emotional awareness” OR “Emotional control” OR “Emotional dysregulation” OR “emotional expressi*” OR “Emotional modulation” OR “Emotional regulation” OR “Mood”) AND (“Obesity” OR “Overweight”) AND (“child*”) AND (“Weight control” OR “Weight management” OR “Weight modulation” OR “External eating” OR “Binge eating” OR “Affective eating” OR “Body mass index” OR “BMI” OR “Eating attitude” OR “Eating behavior” OR “Eating habit” OR “Eating practice” OR “Eating style” OR “Emotional eating”) NOT “adoles*” NOT “infant”) OR AB = (“Affect regulation” OR “Affect dysregulation” OR “Affect recognition” OR “Alexithymia” OR “Anxiety” OR “Depression” OR “Emotional awareness” OR “Emotional control” OR “Emotional dysregulation” OR “emotional expressi*” OR “Emotional modulation” OR “Emotional regulation” OR “Mood”) AND (“Obesity” OR “Overweight”) AND (“child*”) AND (“Weight control” OR “Weight management” OR “Weight modulation” OR “External eating” OR “Binge eating” OR “Affective eating” OR “Body mass index” OR “BMI” OR “Eating attitude” OR “Eating behavior” OR “Eating habit” OR “Eating practice” OR “Eating style” OR “Emotional eating”)

**Table 2:** Study characteristics.

Authors	Year	Country	Study Aim	Sample			Instruments	
				*n*	Age Range M (SD)	Gender	Emotion/Affect	Eating Habits
Farrow et al. [50]	2011	UK	Explore the similarities between unhealthy weight and shape related attitudes and behaviors in a sample of 8–11-year males and females, and evaluate whether individual levels of anxiety are related to children’s susceptibility to peer influences on dysfunctional eating.	154	8–11 10.47 (0.88)	78 girls 75 boys	SCAS	EPI-C
Holt and Ricciardelli [52]	2002	Australia	Examine the utilization of social comparisons and negative affect as indicators of body dissatisfaction and problem eating, exercising, and muscle concerns for boys and girls separately.	236	8–10 Girls = 8.89 (0.69) Boys = 8.94 (0.72)	69 girls 47 boys	PANAS-C	ChEAT
Houldcroft et al. [51]	2014	UK	Examine the potential relationship between preadolescents’ perceptions of controlling parental feeding practices and their reports of anxiety and depression symptomology.	356	7.25–10.25 8.75 (0.57)	172 girls 184 boys	SCAS CDI:S	EPI-C KCFQ
Kirk et al. [56]	2015	Canada	Explore the relationship between food security, diet quality, weight status, and psychosocial outcomes.	5853	10–11	–	Ten questions created ad hoc	US HFSSM DQI YAQ Canadian Nutrient File
Morgan et al. [53]	2002	USA	Investigate self-reported binge eating behaviors in overweight children.	112	6–10	60 girls 52 boys	CDI STAIC	ChEAT
Saling et al. [54]	2005	Australia	Investigate whether low self-esteem, perceived parent and peer relations, negative affect and/or perfectionism predicted dieting, food preoccupation or muscle preoccupation are observed in 8–10-year-old preadolescent children over a 10-month period.	326	8–10	150 girls 176 boys	PANAS-C	ChEAT
Tan and Holub [55]	2018	USA	Examine the effects of happiness and sadness on children’s observed snack consumption and their interaction effect on children’s characteristics (i.e., weight, gender, and age).	91	4.5–9 6.8 (1.2)	43 girls 48 boys	Experimental mood induction (three 3-min video clips eliciting happiness, sadness, and neutral emotions)	Observed emotional eating

Note. CDI: Children’s Depression Inventory; CDI:S: Children’s Depression Inventory:Short Version; ChEAT: Children’s Eating Attitudes Test; DQI: Diet Quality Index–International; EPI-C: Eating Pattern Inventory for Children; KCFQ: Kids’ Child Feeding Questionnaire; PANAS-C: Positive and Negative Affect Schedule for Children; SCAS: Spence Children’s Anxiety Scale; STAIC: State-Trait Anxiety Inventory for Children, A-Trait scale; US HFSSM: Household Food Security; YAQ: Youth Adolescent Food Frequency Questionnaire.

## Data Availability

The data that support the findings of this study are available on request from the corresponding author [S.C.].

## Supplementary Materials

The following supporting information can be downloaded at https://www.mdpi.com/article/10.3390/nu15153343/s1, Table S1: Study characteristics.
