# Supplementary material for: Affect, Body, and Eating Habits in Children: A Systematic Review
Source: Nutrients. 2023 Jul 27;15(15):3343. doi: 10.3390/nu15153343 (PMC10420931; doi:10.3390/nu15153343)
Supplement: Supplementary file 1 [file nutrients-15-03343-s001.zip › nutrients-2511332-supplementary.pdf]

Table S1: Mixed Methods Appraisal Tool (MMAT).

Authors	Screening questions		Category of study designs									
			2. Quantitative randomized controlled trials					4. Quantitative descriptive				
	S1	S2	2.1	2.2	2.3	2.4	2.5	4.1	4.2	4.3	4.4	4.5
Farrow et al.	Y	Y						Y	Y	Y	C	Y
Holt & Ricciardelli	Y	Y						Y	Y	Y	C	Y
Houldcroft et al.	Y	Y						Y	Y	Y	C	Y
Kirk et al.	Y	Y						Y	Y	Y	Y	Y
Morgan et al.	Y	Y						Y	Y	Y	Y	Y
Saling et al.	Y	Y						Y	Y	Y	Y	Y
Tan & Holub	Y	Y	C	Y	Y	C	Y					

Note: Y = Yes; C = Can't tell
